# Statement of Retraction: Long non-coding RNA 00960 promoted the aggressiveness of lung adenocarcinoma via the miR-124a/SphK1 axis

**DOI:** 10.1080/21655979.2024.2299531

**Published:** 2024-02-20

We, the Editors and Publisher of the journal Bioengineered, have retracted the following article:

Zhipeng Ge, Haibo Liu, Tao Ji, Qiaoling Liu, Lulu Zhang, Pengchong Zhu, Liang Li & Liangming Zhu (2022) Long non-coding RNA 00960 promoted the aggressiveness of lung adenocarcinoma via the miR-124a/SphK1 axis, Bioengineered, 13:1, 1276-1287, DOI: 10.1080/21655979.2021.1996507

Since publication, significant concerns have been raised about the integrity of the data and reported results in the article. When approached for an explanation, the authors did not provide their original data or any necessary supporting information. As verifying the validity of published work is core to the integrity of the scholarly record, we are therefore retracting the article. All authors listed in this publication have been informed.

We have been informed in our decision-making by our editorial policies and the COPE guidelines.

The retracted article will remain online to maintain the scholarly record, but it will be digitally watermarked on each page as ‘Retracted’.

